# Residential Greenness and Birth Outcomes: Evaluating the Influence of Spatially Correlated Built-Environment Factors

**DOI:** 10.1289/ehp.1308049

**Published:** 2014-07-11

**Authors:** Perry Hystad, Hugh W. Davies, Lawrence Frank, Josh Van Loon, Ulrike Gehring, Lillian Tamburic, Michael Brauer

**Affiliations:** 1 College of Public Health and Human Sciences, Oregon State University, Corvallis, Oregon, USA; 2 School of Population and Public Health, and; 3 School of Community and Regional Planning, University of British Columbia, Vancouver, British Columbia, Canada; 4 Institute for Risk Assessment Sciences, Utrecht University, Utrecht, the Netherlands

## Abstract

Background: Half the world’s population lives in urban areas. It is therefore important to identify characteristics of the built environment that are beneficial to human health. Urban greenness has been associated with improvements in a diverse range of health conditions, including birth outcomes; however, few studies have attempted to distinguish potential effects of greenness from those of other spatially correlated exposures related to the built environment.

Objectives: We aimed to investigate associations between residential greenness and birth outcomes and evaluate the influence of spatially correlated built environment factors on these associations.

Methods: We examined associations between residential greenness [measured using satellite-derived Normalized Difference Vegetation Index (NDVI) within 100 m of study participants’ homes] and birth outcomes in a cohort of 64,705 singleton births (from 1999–2002) in Vancouver, British Columbia, Canada. We also evaluated associations after adjusting for spatially correlated built environmental factors that may influence birth outcomes, including exposure to air pollution and noise, neighborhood walkability, and distance to the nearest park.

Results: An interquartile increase in greenness (0.1 in residential NDVI) was associated with higher term birth weight (20.6 g; 95% CI: 16.5, 24.7) and decreases in the likelihood of small for gestational age, very preterm (< 30 weeks), and moderately preterm (30–36 weeks) birth. Associations were robust to adjustment for air pollution and noise exposures, neighborhood walkability, and park proximity.

Conclusions: Increased residential greenness was associated with beneficial birth outcomes in this population-based cohort. These associations did not change after adjusting for other spatially correlated built environment factors, suggesting that alternative pathways (e.g., psychosocial and psychological mechanisms) may underlie associations between residential greenness and birth outcomes.

Citation: Hystad P, Davies HW, Frank L, Van Loon J, Gehring U, Tamburic L, Brauer M. 2014. Residential greenness and birth outcomes: evaluating the influence of spatially correlated built-environment factors. Environ Health Perspect 122:1095–1102; http://dx.doi.org/10.1289/ehp.1308049

## Introduction

More than half of the world’s population now live in urban environments, and it has been estimated that by 2050 this number will grow to 60% (approximately 6.4 billion people) (World Health Organization 2013). A diverse range of characteristics associated with living in urban environments—ranging from environmental hazards to social support to health care services—are important to health (Vlahov et al. 2007). Recently, a growing body of evidence has linked exposure to urban greenness (also referred to as green space or natural environments) with measures of health, including mortality (Donovan et al. 2013; Mitchell and Popham 2008; Takano et al. 2002; Villeneuve et al. 2012), respiratory illness (Villeneuve et al. 2012), well-being (Groenewegen et al. 2006; Lafortezza et al. 2009; Maas et al. 2006), and mental health (Sugiyama et al. 2008; Van den Berg et al. 2010; Ward Thompson et al. 2012).

Only a few studies have examined associations between exposure to residential greenness during pregnancy and birth outcomes. Adverse birth outcomes, such as preterm birth and low birth weight, are important not only because of their immediate impacts on infant health but also because of the subsequent health and developmental consequences through the individual’s life course (Blumenshine et al. 2010). In Portland, Oregon, a study of 5,295 births observed that a 10% increase in tree-canopy cover within 50 m of a residence was associated with a significant decrease in small for gestational age (SGA) births [odds ratio (OR) = 0.85; 95% CI: 0.76, 0.94], with no association observed for preterm births (Donovan et al. 2011). A study of 2,393 births from four Spanish cohorts observed similar relationships (Dadvand et al. 2012c). An interquartile range (IQR) increase in average greenness [assessed using satellite-based Normalized Difference Vegetation index (NDVI)] within 500 m of residences was associated with an increase in birth weight of 44.2 g (95% CI: 20.2, 68.2) and an increase in head circumference of 1.7 mm (95% CI: 0.5, 2.9). No associations were observed with measures of gestational age. In another cohort of 8,246 births in Barcelona, Spain, NDVI within 100 m of residences was not associated with birth weight or gestational age in the entire cohort; but in the group with the lowest educational attainment, increasing greenness was associated with higher birth weight (Dadvand et al. 2012a). Finally, for 3,203 births in Munich, Germany, between 1996 and 1999, an IQR increase in greenness within 500 m of residences was associated with a 17.6-g (95% CI: 0.5, 34.6) higher mean birth weight (Markevych et al. 2014).

Given this suggestive evidence and the large potential burden accompanying adverse birth outcomes, it is important to evaluate the robustness of the association between greenness and pregnancy outcomes and the specific pathways through which potential effects may operate. In particular, there is a need to distinguish the effect of residential greenness from other spatially correlated built-environment factors. Here we define built environment as urban design, land use, and the transportation system, encompassing patterns of human activity within the physical environment (Handy et al. 2002). There are four general pathways by which we hypothesize greenness may influence birth outcomes: 1) through the reduction of harmful environmental exposures such as air and noise pollution (e.g., Dadvand et al. 2012b); 2) by providing space for increased utilitarian and recreational physical activity (e.g., Sugiyama et al. 2008); 3) by providing a setting for psychosocial influences, such as increased social contacts and community belonging (e.g., Fan et al. 2011); and 4) through directly reducing psychological stress and depression (e.g., Ward Thompson et al. 2012). Here we focused on evaluating the pathways related to reductions of environmental exposures and increases in physical activity levels (pathways 1 and 2, above).

Reduction of air pollution and noise exposures may be a pathway by which residential greenness influences pregnancy outcomes, due partly to the potential spatial clustering of these exposures. Ambient air pollution can vary dramatically within urban areas (Jerrett et al. 2005), and higher exposure during pregnancy has been associated with low birth weight, preterm birth, small for gestational age, and intrauterine growth retardation (Brauer et al. 2008; Padula et al. 2012; Shah and Balkhair 2011; Srám et al. 2005). Increased exposure to air pollution among pregnant women has been associated with decreased residential greenness (Dadvand et al. 2012b). In the four Spanish birth cohorts study, the effect size for greenness on birth weight was slightly attenuated (from 36.1 to 28.5 g per IQR increase in NDVI) after the inclusion of nitrogen dioxide (NO_2_) exposures (Dadvand et al. 2012c). No study has included noise exposure, which we (Gehring et al. 2014) and others (Knipschild et al. 1981; Schell 1981) have shown may be associated with preterm birth and birth weight. Whether noise and residential greenness levels are spatially correlated is unknown.

Physical activity is another important pathway through which residential greenness may influence birth outcomes (Hegaard et al. 2007). Although proximity to green space as an indicator of physical activity resources and activity levels has been widely used in the literature (e.g., Kaczynski and Henderson 2007), more generalized measures of the built environment (referred to as walkability here) are increasingly found to predict levels of physical activity and are suggested to be an important upstream determinant of behavior (e.g., Durand et al. 2011). Walkability is a composite index of the built environment that is associated with physical activity (Frank et al. 2010) and may also be associated with residential greenness levels.

Here we used a large population-based birth cohort of 64,705 singleton births (from 1999–2002) in Vancouver, British Columbia, Canada, to examine associations between residential greenness and birth outcomes. We also evaluated the extent to which these associations may be mediated by additional spatially correlated factors related to the built environment (air pollution, noise, walkability of residential neighborhoods, and access to parks) to help elucidate potential pathways of influence.

## Methods

*Description of cohort*. Linked administrative data were used to establish a birth cohort that comprised all births from the period 1999–2002 in the metropolitan area of Vancouver (Brauer et al. 2008). Medical services and hospitalization data were provided by the BC (British Columbia) Ministry of Health (2009a, 2009b); vital statistics data by Population Data BC (2009); and perinatal data by Perinatal Services BC (2009). A total of 82,347 births were identified over the 4 years (1999–2002), and 73,387 had mothers with verified complete residential history information within the study area during the 9 months of pregnancy. We excluded 988 multiple births, 7 children without recorded birth weight or parity status, 2,014 births to women with missing maternal age, 1,593 children with missing First Nations status (ethnicity), and 1,197 who were missing specific census covariates. The cohort was therefore reduced to 68,249 births that had complete covariate information; of these we were able to assign measures of greenness, air pollution, noise pollution, and neighborhood walkability for 64,705 births. The study protocol was approved by the Institutional Review Board (Behavioural Research Ethics Board) of the University of British Columbia (#H04-80161). The British Columbia Ministry of Health, Vital Statistics, and Perinatal Services BC approved access to and use of the data facilitated by Population Data BC for this study.

*Birth outcomes*. All birth outcomes were obtained through vital statistics birth records. We evaluated multiple measures of gestational age: very preterm birth (< 30 weeks gestation) and moderately preterm birth (30–36 weeks gestation) versus term birth (≥ 37 weeks gestation). We examined both very and moderate preterm birth separately to allow focus on the very preterm component, which is of greater clinical relevance. We also evaluated term (≥ 37 weeks of gestation) birth weight and fetal growth (SGA, defined as birth weights below the 10th percentile within the study cohort, stratified by sex, for each week of gestation).

*Covariates*. Individual-level covariates obtained through administrative records included the month and year of birth, infant sex, First Nations status, as well as parity, maternal age, and maternal smoking during pregnancy. No individual-level data were available for income or maternal level of education. We assigned subjects to neighborhood-level income quintiles and maternal education quartiles using census data (Statistics Canada 2001) based on their residence at time of birth. Census dissemination area (DA) level data were used, the smallest geographic areas for which all Canadian Census data are provided and correspond to one or more neighboring blocks with target populations of 400–700 persons (Statistics Canada 2001).

*Exposure assessments*. Exposure to residential greenness, air pollution, noise, neighborhood walkability, and distance to parks were estimated for all residences where mothers lived during the 9-month pregnancy period, geocoded to 6-digit postal codes. We calculated an average exposure across the full period of pregnancy that incorporated time-weighted exposures from each residential address.

*Residential greenness*. We used satellite-derived NDVI to derive a continuous measure of greenness across the study region. NDVI ranges from –1 to 1 (with higher numbers indicating more greenness) based on land surface reflectance of visible (red) and near infrared parts of spectrum (Weier and Herring 2011). To assign greenness measures to study postal codes, we downloaded all cloud-free images from Landsat Enhanced Thematic Mapper Plus (ETM+) (http://landsat.gsfc.nasa.gov/?p=3225) of the Vancouver region for 1999–2002. Average greenness values were extracted for 100- and 250-m areas around residential postal code centroids and both yearly and seasonal (summer, June–August; fall, September–November; winter, December–February; and spring, March–May) greenness values were calculated. We did not assign the 30-m NDVI values to each residential postal code as postal codes typically reflect one side of a city block, and this area is better captured with a 100-m buffer distance. The 30-m NDVI was highly correlated with the 100-m (*r* = 0.80) and 250-m (*r* = 0.69) NDVI measures. The seasonal measures were also highly correlated (*r* > 0.94) with the annual measures, and we therefore present only the annual average NDVI results. Although there were slight differences in the actual NDVI mean values by season [0.17 (winter), 0.29 (summer)], the spatial pattern of NDVI did not change, which therefore resulted in a high correlation with seasonal values. The 100- and 250-m buffer areas were used to assign residential greenness exposure because these distances have been used in prior studies (Dadvand et al. 2012a, 2012c), are at a similar spatial scale to within-city air pollution and noise variation, and have been shown to correspond to expert assessments of residential greenness from detailed visual audits (Rhew et al. 2011).

*Air pollution exposure*. We assessed residential air pollution exposure using high-resolution land use regression (LUR) air pollution models developed for the Vancouver metropolitan area for nitric oxide (NO), nitrogen dioxide (NO_2_), fine particulate matter (≤ 2.5 μm; PM_2.5_), and black carbon (BC). The creation and evaluation of the models has been described previously (Brauer et al. 2008; Henderson et al. 2007; Larson et al. 2009), and such models have been used to examine associations with birth outcomes (Brauer et al. 2008). For the present analysis, we estimated air pollution exposures based on LUR models to capture fine-scale spatial variation in air pollution throughout the study region (which may be spatially correlated with greenness levels), rather than interpolation of fixed-site air pollution monitoring data.

*Noise exposure*. Residential noise exposure was estimated using CadnaA software (http://www.datakustik.com/en/products/cadnaa), a physical model with a focus on transportation-related sources. The creation and evaluation of this model has been described previously (Gan et al. 2012) and used in our previous assessment of noise, air pollution, and birth outcomes (Gehring et al. 2014). Briefly, noise exposure was based on transportation-related information, including road traffic data (e.g., speed limits, traffic volume, fleet composition, and road width), railway data (e.g., type of train, velocity, and frequency), and building heights and footprints. Aircraft noise data were obtained from aircraft noise exposure forecasts produced by Vancouver International Airport Authority (Gan et al. 2012). Noise level estimates were created from these data that integrated noise levels during the day, evening, and nights using average A-weighted equivalent continuous noise levels (24-hr averages) with evening and night exposures penalized by 5 and 10 dB(A), respectively [*L*_den_; described previously by Gan et al. (2012)]. Here we examined road traffic noise exposures (motor vehicle noise) as well as all transportation noise (including motor vehicle, railway and aircraft sources).

*Neighborhood walkability*. A walkability index was used to assess built environment characteristics around residential postal codes that may influence opportunities for physical activity. A detailed description of the methods used to develop the walkability index has been described previously (Frank et al. 2010) as well as associations between walkability and objectively measured physical activity levels (Frank et al. 2005). Briefly, the walkability index captured four measures of neighborhood walkability (residential density, retail floor space–to–land area ratio, land use mix, and street connectivity or intersection density) within a 1-km road network distance around a postal code centroid. High levels of the walkability index represent urban form that encourages walking, whereas low levels represent built environment features that inhibit walking. In addition to the walkability index, straight-line distance to the border of the nearest park was calculated to capture additional potential influences on leisure physical activity opportunities.

*Statistical analysis*. We used logistic and linear regression analyses to estimate associations between residential greenness and birth outcomes. Adjusted analyses included the individual covariates [month and year of birth, sex of the baby, First Nations status (yes/no), parity, maternal age (≤ 19, 20–29, 30–34, 35–40, ≥ 40 years) and maternal smoking during pregnancy (yes/no)] as well as census covariates (maternal education quartiles and income quintiles) described previously. For the term birth weight analyses, a categorical variable for the completed weeks of gestation (range, 22–43) was also included. Associations between greenness exposures and birth outcomes are presented corresponding both to an approximate IQR increase in exposure measures and by quartiles, because cubic regression splines (gam-function in R 2.15.0; R Project for Statistical Computing; http://www.r-project.org) identified slightly nonlinear relationships. Incremental models are presented to evaluate how associations between residential greenness and birth outcomes change when air pollution and noise exposures, neighborhood walkability, and park distance are added. A number of sensitivity analyses were also conducted to determine how associations between residential greenness and birth outcomes changed based on residential mobility during pregnancy, when area contextual factors at the DA level (i.e., low income status, unemployment rate, marital status, family composition variables, building characteristic variables, and visible minority status) were entered into the models, and when models were stratified by area income, education, and visible minority status. Visible minority status is defined as persons, other than Aboriginal peoples, who are non-Caucasian in race or nonwhite in color.

## Results

Descriptive statistics of the overall cohort with complete covariate and exposure information (*n* = 64,705), as well as participants stratified by NDVI quartiles, are summarized in Table 1. A total of 230 births (0.4%) were very preterm (< 30 weeks gestation), 3,189 births (5.0%) were moderately preterm (30–36 weeks), 6,817 births (10.5%) were classified as SGA, and the mean birth weight at full term was 3,483 g. Small differences in birth outcomes as well as individual covariate information can be seen by residential greenness quartiles.

**Table 1:** Characteristics of the Vancouver metropolitan area birth cohort [*n* (%) or mean ± SD] with complete data for greenness and built environment exposures, overall (*n* = 64,705) and according to NDVI quartiles.

Variable	Entire cohort	NDVI Q1(< 0.18)	NDVI Q2(0.18–0.23)	NDVI Q3(0.23–0.29)	NDVI Q4(≥ 0.29)
Study population (*n*)	64,705	15, 316	13,773	18,120	17,466
Birth outcomes
Very preterm birth (< 30 weeks)	230 (0.4)	61 (0.4)	57 (0.4)	63 (0.4)	53 (0.3)
Moderately preterm birth (30–36 weeks)	3,189 (5.0)	824 (5.4)	715 (5.2)	892 (4.9)	758 (4.4)
SGA^*a*^ [*n* (%)]	6,817 (10.5)	1,719 (11.2)	1,572 (11.4)	1,885 (10.4)	1,641 (9.4)
Birth weight (g) at full term^*b*^	3,483 ± 472	3,440 ± 464	3,456 ± 468	3,491 ± 473	3,532 ± 476
Covariates
Female sex	31,341 (48.4)	7,494 (48.9)	6,657 (48.3)	8,707 (48.0)	8,483 (48.6)
Nulliparous	29,339 (45.3)	8,006 (52.3)	6,499 (47.2)	7,926 (43.7)	6,878 (39.4)
Maternal age (years)
≤ 19	957 (1.5)	235 (1.5)	189 (1.4)	289 (1.6)	244 (1.4)
20–29	23,760 (36.7)	5,623 (36.7)	5,213 (37.9)	6,910 (38.1)	6,014 (34.4)
30–34	23,981 (37.1)	5,698 (37.2)	5,013 (36.4)	6,734 (37.1)	6,536 (37.4)
35–40	13,383 (20.7)	3,111 (20.3)	2,815 (20.4)	3,540 (19.5)	3,917 (22.4)
≥ 40	2,624 (4.1)	649 (4.2)	543 (3.9)	677 (3.7)	755 (4.3)
Maternal smoking during pregnancy	4,184 (6.5)	909 (5.9)	742 (5.4)	1,213 (6.7)	1,320 (7.6)
First Nations status	395 (0.6)	146 (1.0)	82 (0.5)	85 (0.4)	95 (0.5)
Maternal education (census quartile)
1st quartile (lowest education)	14,412 (22.3)	3,568 (23.3)	3,298 (24.0)	4,332 (23.9)	2,809 (18.2)
2nd quartile	14,654 (22.7)	3,149 (20.6)	3,336 (24.2)	4,208 (23.2)	3,402 (22.0)
3rd quartile	17,669 (27.3)	3,690 (24.1)	3,815 (27.7)	5,152 (28.4)	4,450 (28.8)
4th quartile (highest education)	17,970 (27.8)	4,909 (32.1)	3,324 (24.1)	4,458 (24.6)	4,772 (30.9)
Income (census quintile)					
1st quintile (lowest income)	13,815 (21.4)	5,823 (38.0)	3,603 (26.2)	2,929 (16.2)	1,450 (8.3)
2nd quintile	14,377 (22.2)	3,791 (24.8)	3,597 (26.1)	4,092 (22.6)	2,897 (16.6)
3rd quintile	13,666 (21.2)	2,413 (15.8)	3,012 (21.9)	4,326 (23.8)	3,915 (22.4)
4th quintile	12,524 (19.4)	1,753 (11.5)	2,095 (15.2)	4,086 (22.5)	4,590 (26.3)
5th quintile (highest income)	10,323 (16.0)	1,536 (10.0)	1,466 (10.6)	2,707 (14.9)	4,614 (26.4)
Change of address during pregnancy	21,990 (34.0)	5,129 (33.5)	5,649 (41.0)	6,685 (36.8)	4,389 (28.4)
^***a***^Birth weight below the 10th percentile of the cohort, stratified by sex, for each week of gestation. ^***b***^≥ 37 weeks of gestation, *n* = 61,286.					

**Figure 1:**
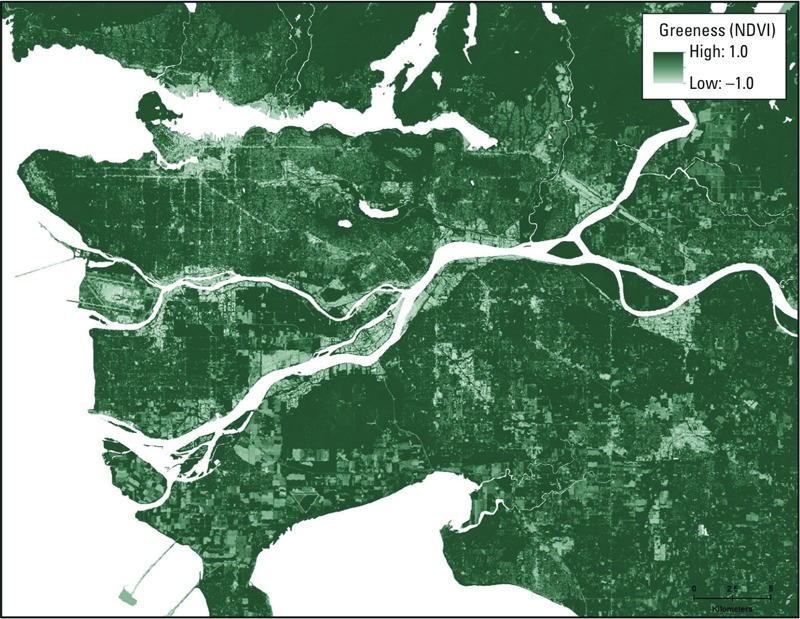
Spatial distribution of greenness for the study cohort measured with satellite-derived NDVI, Vancouver.

**Figure 2:**
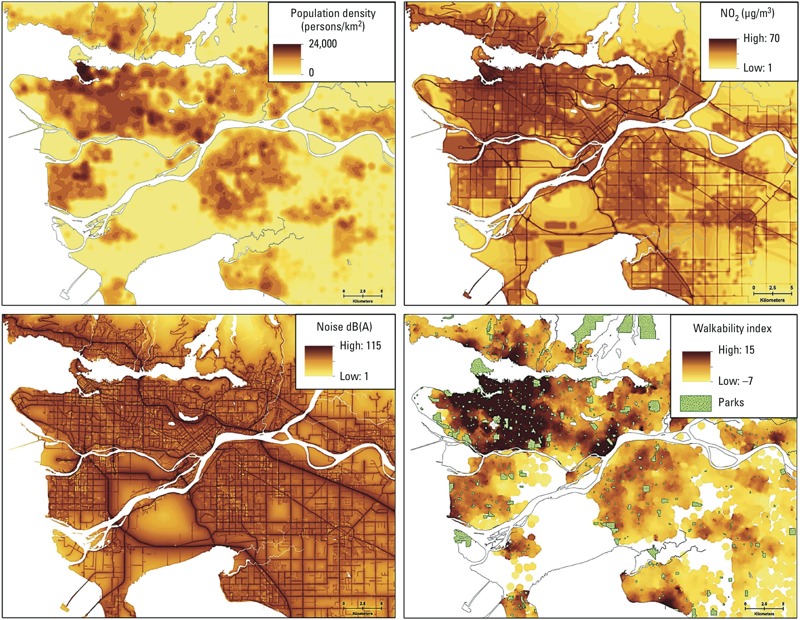
Spatial distribution of study population and annual NO_2_ air pollution concentrations, all noise levels, and neighborhood walkability and park locations, Vancouver.

Table 2 summarizes the distribution of residential greenness exposures as well as other built environment exposure variables. The mean annual residential greenness value (using the NDVI) was 0.24. The spatial distribution of NDVI values for the study area is shown in Figure 1. The correlations between residential greenness exposure and other spatially derived exposure variables are presented in Supplemental Material, Table S1. Low to moderately negative correlations were observed between average NDVI levels within 100 m of residences and exposure to NO (–0.43), NO_2_ (–0.42), PM_2.5_ (–0.36) and BC (–0.31), exposure to traffic (–0.05) and all noise (–0.20), and neighborhood walkability (–0.58) and distance to the nearest park (–0.05). Figure 2 illustrates the spatial distributions of the study population as well as NO_2_ air pollution concentrations, all noise levels, and neighborhood walkability and parks. For reference, the PM_2.5_ exposure map is shown in Supplemental Material, Figure S1. The spatial autocorrelation of these exposure variables within the study area was measured by the Global Moran’s *I* spatial statistic (ESRI ArcGIS 10.1; inverse distance spatial weights matrix and 1 km threshold distance). The resulting spatial autocorrelation for exposure variables were as follows: NDVI (*I* = 2.81), NO (*I* = 0.06), NO_2_ (*I* = 0.04), PM_2.5_ (*I* = 0.03), and BC (*I* = 0.03), neighborhood walkability (*I* = 0.92), park proximity (*I* = 0.38), road traffic noise (*I* = 0.47), and all transportation noise (*I* = 0.48). These spatial autocorrelation measures were sensitive to the spatial weights and distance thresholds used, with increasing autocorrelation generally seen for larger distances (data not shown).

**Table 2:** Summary of residential greenness and other spatially derived environmental exposure variables for the birth cohort (*n* = 64,705).

Exposure	Mean ± SD	Minimum	P25	P50	P75	Maximum
Greenness
NDVI Index	0.24 ± 0.08	–0.08	0.18	0.24	0.28	0.59
Air pollution
NO–LUR (μg/m^3^)	31.5 ± 3.8	1.4	22.5	28.3	37.2	149.6
NO_2_–LUR (μg/m^3^)	33.7 ± 9.1	0.0	27.4	31.9	36.9	64.5
PM_2.5_–LUR (μg/m^3^)	4.1 ± 1.7	0.0	3.2	4.0	4.7	11.3
BC–LUR (10^–5^/m)^*a*^	1.6 ± 1.2	0.0	0.9	1.0	2.0	5.4
Noise [dB(A)]
Traffic noise	60.2 ± 5.3	6.2	57.0	59.9	63.1	89.0
All noise	61.6 ± 5.2	6.2	58.5	61.1	64.5	94.7
Neighborhood walkability
Walkability index	0.37 ± 3.07	–7.80	–1.80	–0.40	2.55	13.20
Park distance (m)	422 ± 383	1	200	325	525	5,850
P25, P50, and P75 are 25th, 50th, and 75th percentiles. ^***a***^Black carbon, based on the particle light absorption coefficient, was highly correlated with the concentrations of elemental carbon measured by traditional thermal/optical reflectance (*R*^2^ = 0.7–0.8); 10^–5^/m black carbon is approximately equivalent to 0.8 μg/m^3^ elemental carbon (Rich 2002).						

The associations between residential greenness and other built environment exposure measures and birth outcomes are shown in Table 3. These estimates represent each variable entered into separate models (i.e., not mutually adjusted exposure models). For an IQR increase (0.1) in NDVI, the OR for very preterm birth was 0.91 (95% CI: 0.77, 1.07), for moderately preterm birth was 0.95 (95% CI: 0.91, 0.99), and for SGA was 0.97 (95% CI: 0.94, 1.00). Term birth weight was 20.6 g (95% CI: 16.5, 24.7 g) higher on average with each 0.1 increase in NDVI. When individuals with the highest quartile of NDVI values (> 0.29) were compared with the lowest (< 0.18), the ORs for very and moderately preterm birth and small for gestational age were 0.80 (95% CI: 0.55, 1.18), 0.87 (95% CI: 0.79, 0.96), and 0.95 (95% CI: 0.88, 1.03), respectively. Term birth weight was also 44.6 g (95% CI: 34.8, 54.4 g) higher for the highest NDVI quartile compared with the lowest. As previously reported, air pollution was associated with very preterm birth and noise and air pollution with SGA and term birth weight (Brauer et al. 2008; Gehring et al. 2014). Higher levels of neighborhood walkability were associated with adverse birth outcomes, a finding that may reflect the positive correlation between walkability and air pollution exposures previously reported for the metropolitan Vancouver area (Marshall et al. 2009).

**Table 3:** ORs (95% CIs) for preterm birth and SGA, and average difference in birth weight based on separate models of associations with greenness and other spatially derived built environment exposure variables.

Exposure	Very preterm birth (< 30 weeks) OR (95% CI)	Moderately preterm birth(30–36 weeks) OR (95% CI)	SGAOR (95% CI)	Term birth weight β (95% CI)
Greenness, NDVI 100 m
Per 0.1 unit	0.91 (0.77, 1.07)	0.95 (0.91, 0.99)	0.97 (0.94, 1.00)	20.6 (16.5, 24.7)
Q1 (< 0.18)
Q2 (0.18–0.23)	0.94 (0.66, 1.33)	0.95 (0.86, 1.05)	1.06 (0.99, 1.13)	3.2 (–6.2, 12.7)
Q3 (0.24–0.28)	0.85 (0.59, 1.24)	0.95 (0.86, 1.06)	1.01 (0.94, 1.08)	19.2 (9.4, 29.0)
Q4 (≥ 0.29)	0.80 (0.55, 1.18)	0.87 (0.78, 0.96)	0.95 (0.88, 1.03)	44.6 (34.8, 54.4)
Air pollution
NO–LUR, per 10 μg/m^3^	1.00 (0.91, 1.11)	1.01 (0.99, 1.04)	1.02 (1.00, 1.04)	–6.5 (–9.1, –3.9)
NO_2_–LUR, per 10 μg/m^3^	1.05 (0.91, 1.22)	1.02 (0.98, 1.06)	0.98 (0.96, 1.01)	–5.2 (–9.1, –1.4)
PM_2.5_–LUR, per 1 μg/m^3^	1.07 (1.00, 1.15)	1.01 (0.99, 1.03)	1.01 (0.99, 1.02)	–3.1 (–5.1, –1.1)
BC–LUR, per 10^–5^/m	0.97 (0.87, 1.08)	1.00 (0.98, 1.03)	1.02 (1.00, 1.04)	–3.4 (–6.2, –0.6)
Noise
All noise, per 6 dB(A)	1.00 (0.86, 1.16)	1.03 (0.99, 1.07)	1.10 (1.06, 1.13)	–19.1 (–22.9, –15.3)
Traffic noise, per 6 dB(A)	0.97 (0.84, 1.12)	1.02 (0.98, 1.06)	1.09 (1.06, 1.12)	–16.8 (–20.5, –13.1)
Neighborhood walkability
Walkability index, per 4 units	1.06 (0.89, 1.27)	1.04 (1.00, 1.09)	1.00 (0.96, 1.03)	–12.6 (–17.2, –8.0)
Distance to park, per 300 m	1.01 (0.91, 1.12)	0.97 (0.94, 1.00)	0.97 (0.95, 0.99)	5.3 (2.7, 7.9)
Q, quartile. Adjusted for sex, parity, First Nations status, maternal age, maternal smoking during pregnancy, maternal education, income, and year and month of birth; term birth weight was additionally adjusted for completed weeks of gestation.				

**Figure 3:**
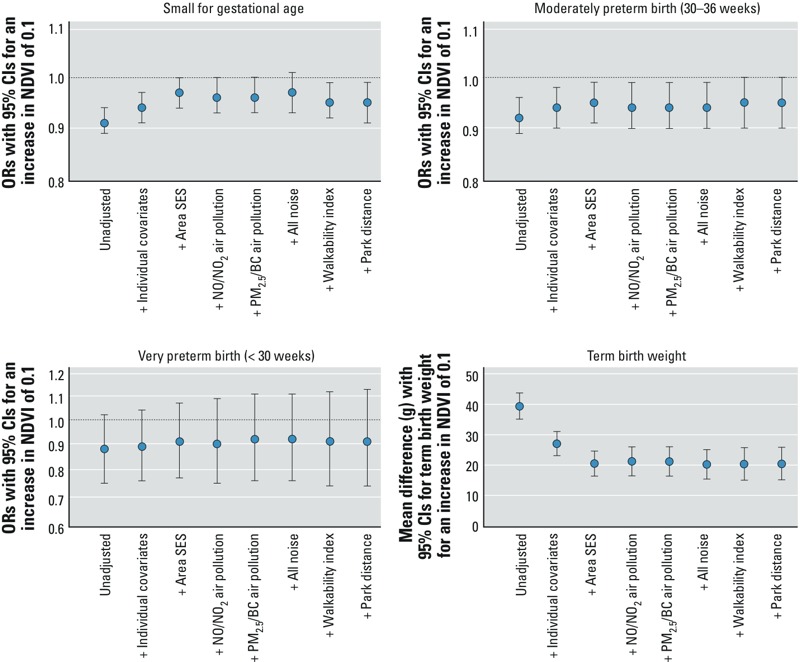
Incremental models of exposure to residential greenness and birth outcomes (note changing scale of *y*-axis). Models including individual + area SES (3rd bar) are shown in Table 3. Models at the far right (+ Park distance) are fully adjusted models including individual covariates, area SES measures, and all built environment exposures.

We used incremental models to examine how the associations between residential greenness and birth outcomes changed with the inclusion of the other built environment exposure variables. Figure 3 illustrates that the inclusion of individual and area socioeconomic status (SES) indicators attenuated the associations of residential greenness with moderately preterm birth, SGA, and term birth weight, whereas the inclusion of air pollution, noise, walkability, and park distance had relatively little influence. Individual and area SES indicators were entered first to control for potential confounding factors. The order that environmental variables were entered into the model did not change observed associations. In the fully adjusted model that included all built environment exposure variables, an IQR increase of 0.1 in NDVI was associated with ORs for very preterm birth, moderate preterm birth, and SGA of 0.91 (95% CI: 0.74, 1.13), 0.95 (95% CI: 0.90, 1.00), and 0.95 (95% CI: 0.91, 0.99), respectively, and term birth weight was 19.5 g (95% CI: 14.1, 24.9 g) higher on average. An illustration of how individual coefficients for environmental variables and birth weight changed during adjustments is shown in Supplemental Material, Table S2. In general, the associations between air pollution, neighborhood walkability, and park distance with birth weight largely disappeared with the inclusion of greenness exposures, and the effects of noise exposures were reduced by approximately 50%. In a fully adjusted model including all built environment exposure variables, associations with birth weight other than greenness were highly variable, perhaps due to multicollinearity. In particular, the two noise exposure variables are highly correlated (*r* = 0.92), and the individual variable coefficients are therefore difficult to interpret. Similar patterns were seen for preterm birth, moderate preterm birth, and SGA (data not shown).

Smooth exposure–response curves for residential greenness and birth outcomes from the unadjusted, adjusted, and fully adjusted models (including other built environment variables) are shown in Supplemental Material, Figure S2. For all outcomes there is a statistically significant nonlinear relationship (*p* < 0.01 for linear versus nonlinear spline function), with an inflection point occurring around the NDVI value of 0.15, after which the influence of residential greenness on birth outcomes becomes positive and linear. A map of NDVI values above and below this threshold is shown in Supplemental Material, Figure S3.

*Sensitivity analyses*. In this birth cohort, 36.9% of women moved residence during their pregnancy. We found little difference in residential mobility between women currently living in the lowest area income quintile who moved (36.9% of women) compared with women currently living in the highest income quintile who moved (37.3%). When the fully adjusted models were stratified by moving status, only slight differences were observed between movers and nonmovers (data not shown). For example, in movers a 0.1 increase in NDVI was associated with an increase in term birth weight of 20.1 g (95% CI: 12.3, 28.0 g), compared to 21.9 g (95% CI: 17.0, 26.7 g) for nonmovers.

In place of detailed individual characteristics, we also explored a number of additional area-level variables (e.g., low income status, unemployment rate, marital status, family composition variables, building characteristic variables, and visible minority status) to determine their influence on associations between greenness and birth outcomes. Associations were robust to inclusion of all area-level variables except for those related to the proportion of visible minorities, which attenuated results, primarily for term birth weight (data not shown). For example, including percent Chinese or percent South Asian ethnicity by DA areas [the two most prominent visible minority groups in the study area, who also have lower birth weight (Janssen et al. 2007)] reduced the association between NDVI and term birth weight from 20.6 g (95% CI: 16.5, 24.7 g) to 14.0 g (95% CI: 9.4, 18.6 g) per 0.1 increase in NDVI. These results need to be interpreted with caution, however, because visible minority groups were clustered within the study region, and these variables may act as a proxy for geographic area. This may therefore underestimate the true effects of residential greenness due to reductions in the spatial contrast and variation of exposures. In addition, although Chinese and South Asian babies have similarly lower normal birth weights compared with the general population in this study area (Janssen et al. 2007), we found that the above attenuation was driven by the Chinese classification, and inclusion of only the South Asian classification increased the strength of associations between NDVI and birth weight (data not shown).

Models stratified by area income, education, and percent Chinese or percent South Asian were explored to further examine potential spatial and residual confounding (see Supplemental Material, Table S4). Associations were slightly stronger in the highest income quintile, especially for term birth weight where a 0.1 increase in NDVI was associated with a 32.4-g (95% CI: 20.3, 44.5) higher average birth weight. However, with stratificaiton by area-level education and visible minority status, no clear gradients for any birth outcome were observed.

## Discussion

We found that increased greenness within 100 m of residences measured using satellite-derived NDVI was associated with higher term birth weight and with reduced likelihood of very (< 30 weeks) and moderately (30–36 weeks) preterm births and SGA. These associations were robust to adjustment for air pollution and noise exposures, the walkability of residential neighborhoods, and distance to the nearest park. This suggests that the association between greenness and birth outcomes is independent of these spatially varying exposures related to the built environment, and that alternative pathways may link residential greenness to birth outcomes.

Our results are consistent with those of the small number of studies that have examined residential greenness and birth outcomes. Recalculated for the NDVI increment of 0.1 used in our study, the study of four Spanish cohorts estimated that birth weight was 22.3 g higher in association with a 0.1-unit increase in average NDVI within 100 m (Dadvand et al. 2012c), which is very similar to the estimated effect for our study population. Adjusting for NO_2_ air pollution exposures in the Spanish study reduced the magnitude of the association with NDVI within 100 m by 21% to 17.7 g. In the cohort study of 3,203 births in Munich, the strongest association was observed for NDVI within 500 m. For NDVI within 100 m (the metric used in our study), adjusting for NO_2_ exposure increased the estimated difference in birth weight from 12.2 g to 16.2 g (Markevych et al. 2014). Adjustment for PM_2.5_ and proximity to major roads also increased the magnitude of effect estimates for NDVI, whereas adjusting for noise attenuated the NDVI and birth weight association by 56%. There were differences in associations estimated for NDVI within 100-, 250-, 500- and 800-m buffer areas in the Munich study, especially once adjusted for air pollution and noise exposures. In our study, we observed no appreciable attenuation of NDVI with adjustment for air pollution, noise, neighborhood walkability, and the distance to the nearest park. In the cohort study of 8,246 births in Barcelona, Spain, NDVI within 100 m of homes was associated with birth weight only among the lowest education group (Dadvand et al. 2012b). We did not observe differences between greenness levels within area-level income or education categories (see Supplemental Material, Table S3). The study of 5,295 births in Portland, Oregon, used a different metric of greenness exposure (measured using tree-canopy cover within 50 m of homes), and estimated an OR for SGA of 0.85 (95% CI: 0.76, 0.94) in association with a 10% increase in tree-canopy cover within 50 m (Donovan et al. 2011). Although not directly comparable with our NDVI greenness exposure assessment, our results support these findings. In our study, we also observed inflection points for all birth outcomes at an approximate NDVI value of 0.15 in smoothed exposure–response plots, above which most of the associations between greenness and all birth outcomes were observed. If this relationship can be replicated, it suggests that a certain amount of greenness is required before benefits to birth outcomes (and potentially other health outcomes) are observed. No studies are available to compare with this finding.

The fact that residential greenness exposure remained associated with birth outcomes after adjusting for a number of hypothesized environmental exposures suggests that alternative pathways may link greenness exposure to birth outcomes. Two well-hypothesized pathways that were not examined in this study include psychosocial and psychological influences. Greenness may facilitate positive psychosocial influences by providing shared spaces for interactions. For example, exposure to greenness has been associated with social support (Maas et al. 2009) and increased social ties and community belonging (Kweon et al. 1998; Sugiyama et al. 2008). The psycho-evolutionary model proposed by Ulrich (1983) also suggests a direct biological impact of perceiving the natural environment. For example, exposure to greenness has been associated with reduced blood pressure (Hartig et al. 2003) and heart rate (Ulrich et al. 1991) and with changes in salivary cortisol patterns (Ward Thompson et al. 2012). Although we were unable to examine these pathways, our findings of a persistent greenness association with birth outcomes that was independent of previously observed associations with noise and air pollution suggests that more research is required that includes all potential pathways potentially linking greenness to birth outcomes.

A major strength of our analysis is that, unlike previous studies, we were able to estimate associations between residential greenness and birth outcomes after adjusting for a number of spatially correlated built environment variables. However, our ability to use a large population-based cohort derived from administrative data also incorporates inherent limitations related to the lack of individual covariates. Although a number of individual-level variables were available in these databases, no information was available for individual-level SES [although smoking during pregnancy and maternal age are useful surrogate measures, especially for the lower portion of the SES gradient (BC Ministry of Health 2011) and other determinants of birth outcomes (e.g. maternal diabetes)]. We used the smallest scale of census data available to account for area-level maternal education and income, which attenuated the association between greenness and birth outcomes. Further spatial confounding may be present within the census DA (i.e., correlation between greenness and individual income or education); however, given the fine-scale resolution of the DA census boundary (mean size = 0.4 km^2^) this is unlikely. There was a correlation of –0.30 between residential greenness and percentage of visible minorities, suggesting that spatial confounding may be an issue, although stratified analyses by visible-minority quartiles showed no obvious trend and the largest associations were generally seen for the lowest visible-minority quartile. Although the NDVI measure enabled us to quantify small-scale variations in greenness in a standardized and objective measure, NDVI does not distinguish between different types of vegetation (e.g., trees or grass fields), quality of greenness, or actual exposure to greenness, which may be important depending on the theorized pathways linking greenness to birth outcomes. In addition, the other built environment exposure variables were modeled at different spatial resolutions, which may incorporate differential exposure misclassification. Only residential history during pregnancy was available to assess NDVI, and 9 months represents a relatively short exposure period unless women had lived at their current residential address for longer periods of time. We did not have information on prior residential duration, but we examined residential mobility during pregnancy and found no change in our results. Long-term residential mobility remains a concern if individuals choose to live in neighborhoods based on characteristics that may influence birth outcomes and that are also related to residential greenness.

## Conclusions

Residential greenness represents a unique (and potentially modifiable) exposure construct that may be important to health. We determined that increased residential greenness is associated with positive birth outcomes in a population birth cohort in Vancouver, British Columbia, Canada, and that this association persists after adjusting for exposures to air pollution, noise, residential neighborhood walkability, and park proximity. Further research is needed to evaluate the specific pathways that may link residential greenness exposure to birth outcomes, including other built environment features as well as psychosocial and psychological pathways.

## Supplemental Material

(4.7 MB) PDF
