# Supplementary material for: Residential Greenness and Birth Outcomes: Evaluating the Influence of Spatially Correlated Built-Environment Factors
Source: Environ Health Perspect. 2014 Jul 11;122(10):1095–102. doi: 10.1289/ehp.1308049 (PMC4181932; doi:10.1289/ehp.1308049)
Supplement: (4.7 MB) PDF [file ehp.1308049.s001.508.pdf]

Environmental Health Perspectives doi:10.1289/ehp.1308049

Supplemental Material

Residential Greenness and Birth Outcomes: Evaluating the Influence of Spatially Correlated Built-Environment Factors

Perry Hystad, Hugh W. Davies, Lawrence Frank, Josh Van Loon, Ulrike Gehring, Lillian Tamburic, and Michael Brauer

Table of Contents **Page**

Table S1. Spatial correlation between residential greenness and other built environment exposure variables for all cohort members 2

Table S2. Incremental models for residential greenness and term birth weight when air pollution, noise, neighborhood walkability and park distance are added 3

Table S3. Incremental models for built environment variables and term birth weight when NDVI is added 4

Table S4. Fully adjusted model of residential greenness exposures (linear, per 0.1 unit increase) and pregnancy outcomes stratified by area income, education and visible minority quintiles/quartiles 5

Figure S1. Spatial distribution of $PM_{2.5}$ ($\mu g/m^3$) estimated from a land use regression model 6

Figure S2. Exposure-response plots for residential greenness and birth outcomes. Adjusted models are show in Table 3 and fully adjusted models include all built environment variables 7

Figure S3. Areas with NDVI levels below and above the 0.15 level observed to be influential in exposure-response plots for birth outcomes 8

Table S1. Spatial correlation between residential greenness and other built environment exposure variables for all cohort members.

Variable	NDVI 100 m	NO	NO_2	$PM_{2.5}$	BC	Traffic	All	Walk. index	Park proximity
**Greenness**									
NDVI 100m	1	-0.43	-0.42	-0.36	-0.31	-0.05	-0.20	-0.58	-0.05
**Air pollution**									
NO		1	0.49	0.29	0.55	0.18	0.37	0.41	-0.20
NO_2			1	0.53	0.29	0.05	0.18	0.37	-0.25
$PM_{2.5}$				1	0.17	0.09	0.16	0.26	-0.19
BC					1	0.29	0.37	0.18	-0.10
**Noise**									
Traffic						1	0.92	0.01	-0.08
All							1	0.15	-0.15
**Physical activity**									
Walkability index								1	-0.43
Park proximity									1

Table S2. Incremental models for residential greenness and term birth weight [β (95% CI)] when air pollution, noise, neighborhood walkability and park distance are added.

Greenness, NDVI 100 m	Model 1[a]	+ Air pollution	+ Noise	+ Walkability/ parks	+ All variables
Linear, 0.1	20.6 (16.5, 24.7)	21.3 (16.5, 26.1)	16.4 (12.0, 20.9)	20.6 (15.7, 22.5)	19.5 (14.1, 24.9)
Q1 (< 0.18)	0	0	0	0	0
Q2 (0.18-0.24)	3.2 (-6.2, 12.7)	2.7 (-7.2, 12.7)	-1.3 (-10.9, 8.3)	3.1 (-6.6, 12.8)	1.0 (-9.2, 11.2)
Q3 (0.24-0.29)	19.2 (9.4, 29.0)	18.9 (8.2, 29.5)	12.3 (2.1, 22.4)	18.9 (8.3, 29.5)	16.5 (5.2, 27.8)
Q4 (> 0.29)	44.6 (34.8, 54.4)	44.5 (33.4, 55.6)	34.7 (24.3, 45.2)	43.2 (32.0, 54.5)	39.2 (27.1, 51.4)

[a]Greenness (NDVI) estimates represent models that include all air pollution, noise, neighborhood walkability, and all variables. All models are adjusted for sex, parity, First Nations status, maternal age, maternal smoking during pregnancy, maternal education, income, year and month of birth, and completed weeks of gestation.

Table S3. Incremental models for built environment variables and term birth weight [β (95% CI)] when NDVI is added.

**Built environment variables**	**Model 1[a]**	**+ NDVI**	**+ NDVI**	**+ NDVI**	**+ All variables[b]**
**Air pollution**					
NO – LUR, 10 μg/m^3	-6.5 (-9.1, -3.9)	-0.9 (-3.8, 2.0)			-0.2 (-3.9, 3.5)
NO_2 – LUR, 10 μg/m^3	-5.2 (-9.1, -1.4)	2.4 (1.8, 6.6)			4.8 (-0.2, 9.8)
$PM_{2.5}$ – LUR, 1 μg/m^3	-3.1 (-5.1, -1.1)	0.1 (-1.9, 2.2)			0 (-2.4, 2.4)
BC – LUR, 10^{-5} part./m	-3.4 (-6.2, -0.6)	7.7 (-21.6, 36.9)			4.5 (0.9, 8.1)
**Noise**					
All transportation, 6 dB(A)	-19.1 (-22.9, -15.3)		-10.0 (-13.8, -6.2)		-45.0 (-5.7, -48.6)
Traffic, 6 dB(A)	-16.8 (-20.5, -13.1)		-9.1 (-12.8, -0.5)		31.8 (8.6, 55.1)
**Neighborhood walkability**					
Walkability index, 4	-12.6 (-17.2, -8.0)			0.3 (-5.7, 5.2)	2.1 (-4.0, 8.2)
Distance to nearest park, 300 m	5.3 (2.7, 7.9)			2.6 (-0.1, 1.7)	0.8 (-0.1, 0.2)

[a]Individual exposure variables adjusted for sex, parity, First Nations status, maternal age, maternal smoking during pregnancy, maternal education, income, year and month of birth, and completed weeks of gestation. [b]Model includes NDVI and all built environment exposure variables and sex, parity, First Nations status, maternal age, maternal smoking during pregnancy, maternal education, income, year and month of birth, and completed weeks of gestation.

Table S4. Fully adjusted model of residential greenness exposures (linear, per 0.1 unit increase) and pregnancy outcomes stratified by area income, education and visible minority quintiles/quartiles.

**Area-level variables**	**Very preterm birth (< 30 weeks) OR (95% CI)**	**Moderately preterm birth (30-36 weeks) OR (95% CI)**	**Small for gestational age (OR 95% CI)**	**Term birth weight β (95% CI)**
**Income quintile**				
Q1 (lowest income)	1.02 (0.62, 1.69)	0.99 (0.87, 1.13)	1.03 (0.94, 1.13)	14.8 (2.0, 27.7)
Q2	1.00 (0.67, 1.51)	0.93 (0.82, 1.06)	0.94 (0.86, 1.03)	26.0 (13.9, 38.1)
Q3	0.97 (0.58, 1.60)	0.98 (0.86, 1.12)	0.95 (0.86, 1.04)	14.0 (1.5, 26.5)
Q4	0.61 (0.37, 0.98)	0.99 (0.87, 1.12)	0.90 (0.81, 0.99)	16.1 (4.3, 27.8)
Q5 (highest income)	0.83 (0.49, 1.39)	0.96 (0.83, 1.10)	0.90 (0.82, 1.00)	32.4 (20.3, 44.5)
**Education quartile**				
Q1 (lowest education)	1.16 (0.72, 1.86)	0.92 (0.80, 1.05)	0.93 (0.85, 1.02)	35.6 (22.5, 48.7)
Q2	0.81 (0.51, 1.29)	0.93 (0.82, 1.06)	0.97 (0.89, 1.06)	13.7 (1.8, 25.5)
Q3	0.75 (0.52, 1.10)	0.94 (0.85, 1.05)	1.02 (0.95, 1.10)	13.5 (3.3, 23.6)
Q4 (highest education)	0.83 (0.56, 1.25)	1.05 (0.95, 1.17)	0.88 (0.81, 0.95)	24.7 (15.0, 34.5)
**Percent Chinese**				
Q1 (lowest %)	0.85 (0.56, 1.30)	0.95 (0.84, 1.06)	0.98 (0.90, 1.06)	20.7 (9.7, 31.8)
Q2	0.79 (0.51, 1.20)	0.96 (0.85, 1.07)	0.92 (0.85, 1.00)	17.3 (6.7, 27.9)
Q3	0.86 (0.57, 1.30)	0.84 (0.75, 0.95)	0.91 (0.83, 0.99)	16.5 (5.3, 27.6)
Q4 (highest %)	1.15 (0.74, 1.78)	1.08 (0.95, 1.22)	1.04 (0.96, 1.13)	12.3 (1.0, 23.6)
**Percent South Asian**				
Q1 (lowest %)	0.87 (0.58, 1.31)	0.93 (0.83, 1.04)	0.98 (0.90, 1.06)	13.3 (3.1, 23.6)
Q2	0.71 (0.43, 1.16)	1.01 (0.90, 1.14)	0.94 (0.87, 1.03)	17.5 (6.5, 28.5)
Q3	0.73 (0.49, 1.10)	0.98 (0.87, 1.10)	0.99 (0.91, 1.07)	17.0 (6.4, 27.6)
Q4 (highest %)	1.23 (0.80, 1.90)	0.92 (0.81, 1.05)	0.93 (0.86, 1.02)	24.8 (12.3, 37.3)

Adjusted for sex, parity, First Nations status, maternal age, maternal smoking during pregnancy, area maternal education and income (for other stratified models), year and month of birth, NO, NO_2, $PM_{2.5}$ and BC air pollution exposure, traffic and all noise exposure, neighborhood walkability and distance to nearest parks. Term birth weight was additionally adjusted for completed weeks of gestation.

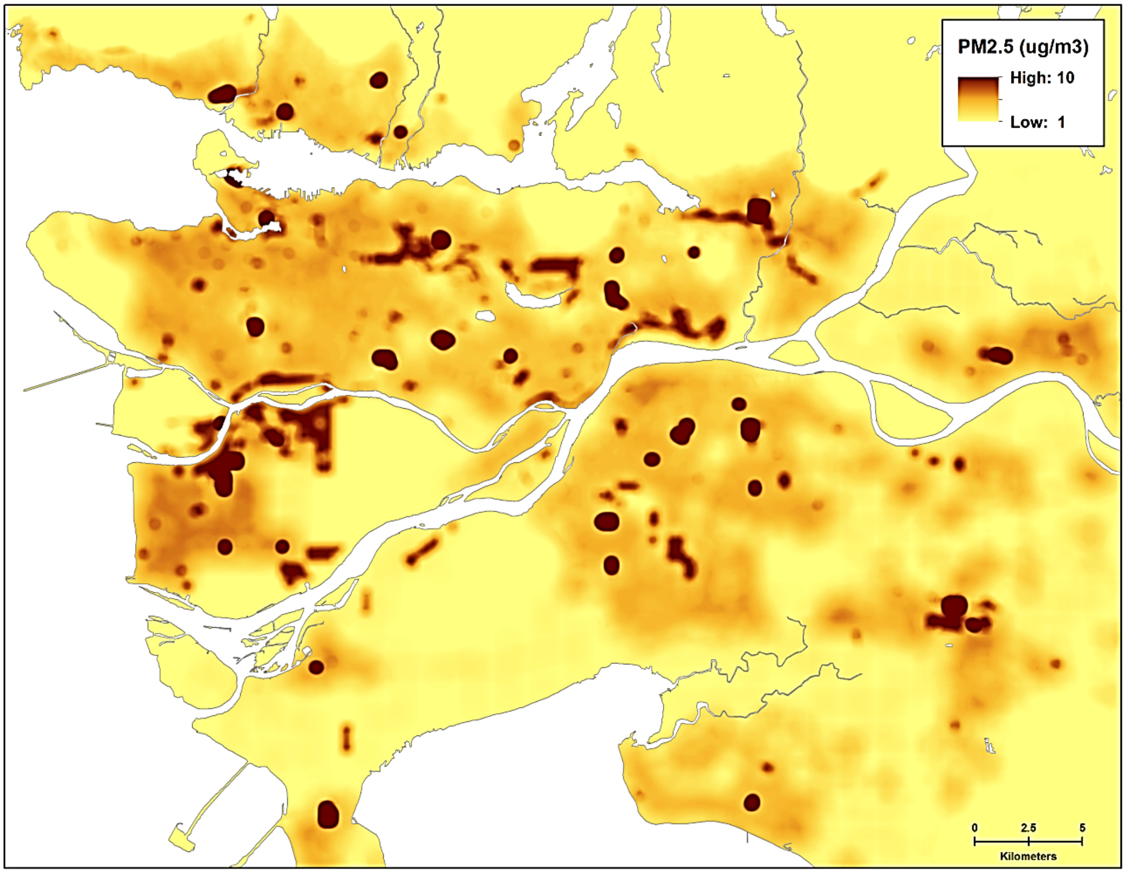

Figure S1. Spatial distribution of $PM_{2.5}$ ($\mu g/m^3$) estimated from a land use regression model.

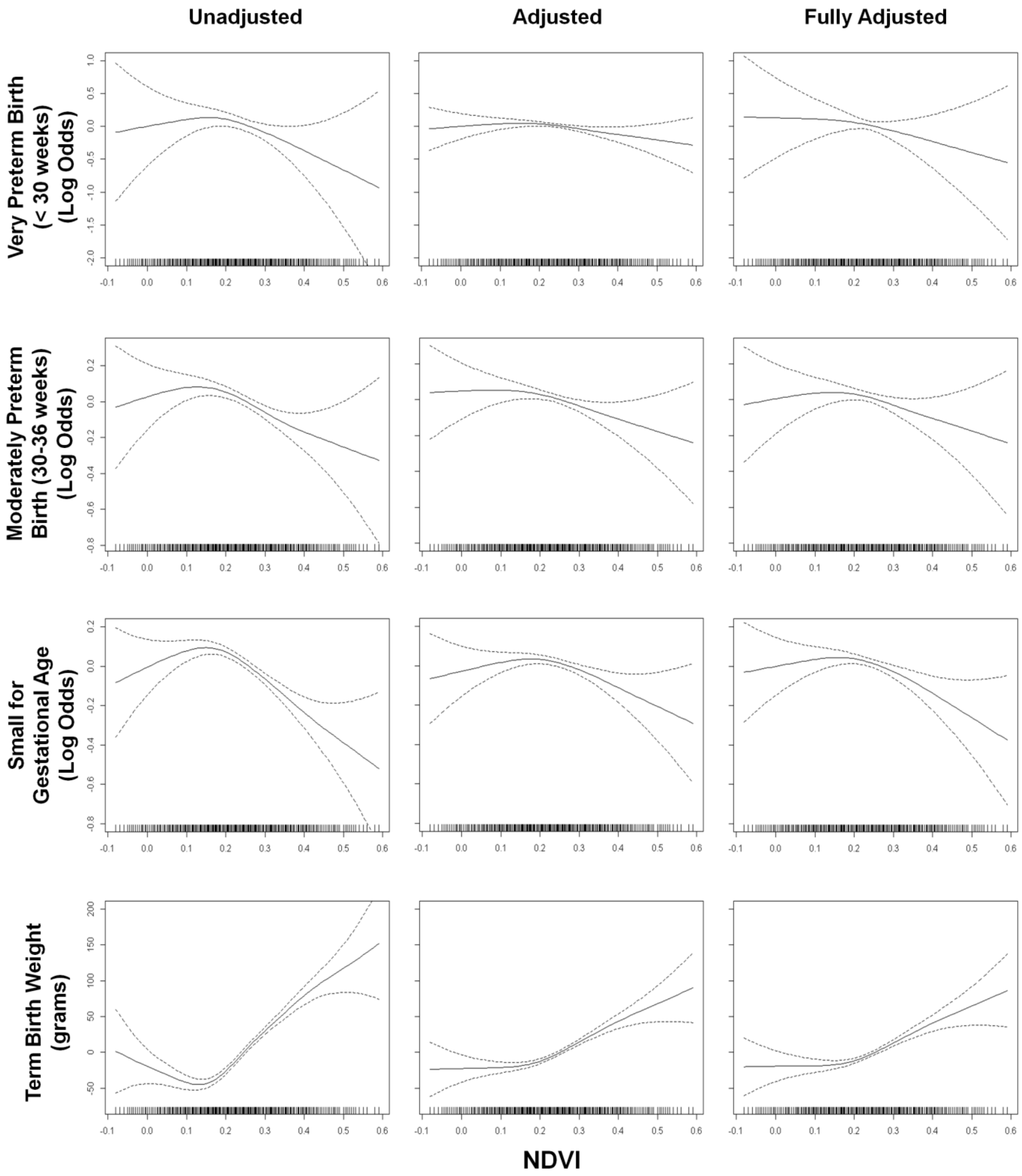

Figure S2. Exposure-response plots for residential greenness and birth outcomes. Adjusted models are show in Table 3 and fully adjusted models include all built environment variables.

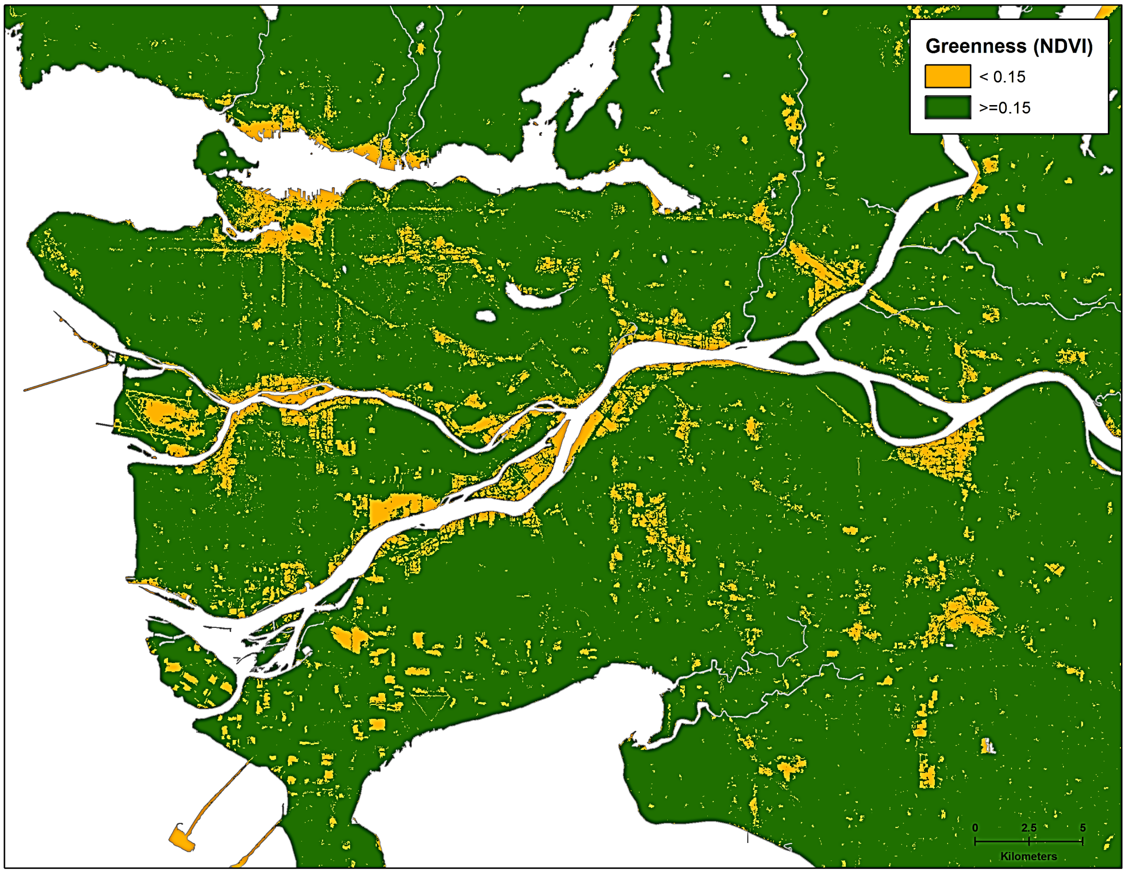

Figure S3. Areas with NDVI levels below and above the 0.15 level observed to be influential in exposure-response plots for birth outcomes.
